# Supplementary material for: Variations in the Human Serum Albumin Gene: Molecular and Functional Aspects
Source: Int J Mol Sci. 2022 Jan 21;23(3):1159. doi: 10.3390/ijms23031159 (PMC8835714; doi:10.3390/ijms23031159)
Supplement: Supplementary file 1 [file ijms-23-01159-s001.zip › ijms-1498010-supplementary.pdf]

Table S1. Genetic variants of human serum albumin

#	Name[a]	Alternative name	Base change[b]	DNA or protein sequencing[c]	Intron/ Exon	Protein change[d]	Notes and functional effects
1	Malmö I	Kaikoura, Tradate, Redhill (see also residue 320), Sweden, Ildut	c.67C>T	D/P	E-1	–2Arg→Cys	Malmö I: 3% proalbumin, 30% Arg-albumin (due to aberrant signal peptide cleavage) with the rest as normal albumin (Alb A). Sweden: 3 homozygotes
2	Lille	Pollibauer, Somalia, Tokushim,Taipei, Fukuoka-2,Varese, Wu Yang, Mayo EW220, Komagone-3, Stirling	c.68G>A	P	E-1	–2Arg→His	
3	Christchurch	Gainesville, Y, Honolulu-2, Fukuoka-3, Mayo JW180, Shizuoka, Kamloops, Zagreb	c.71G>A	P	E-1	–1Arg→ Gln	
4	Takefu	Honolulu-1	c.71G>C	P	E-1	–1Arg→Pro	
5	Jaffna		c.71G>T	P	E-1	–1Arg→ Leu	
6	Blenheim	Bremen, Malmö II, Iowa City-2	c.74A>T	P	E-1	1Asp→ Val	Blenheim: 10% proalbumin, 38% Val-. Bremen: 20% Arg-Alb, 30% Val-. In both cases, the remaining is Alb A. Blenheim:

							decreased α-helical content.
7	Larino		c.79C>T	D/P	E-1	3His→Tyr	Low in vivo stability
8	Nagasaki-3		c.81C>A/G	P	E-2	3His→Gln	
9	Torino		c.250G>A	P	E-3	60Glu→Lys	
10	Dalakarlia-1	Sweden-1, Malmö-95	c.259G>A	D/P	E-3	63Asp→Asn; N-glycosylated at 63Asn	CHO next to a Cys. High thermal stability. Decreased α-helical content
11	FDH-T3		c.269T>C	D	E-3	66Leu→Pro	High T3 binding. Iden tified in a Thai family.
12	Vibo Valentia		c.316G>A	P	E-4	82Glu→Lys	
13	Yanomama-2		c.412C>G	P	E-4	114Arg→Gly	Low bilirubin binding
14	Nagoya		c.427G>A	P	E-4	119Glu→Lys	
15	Tregasio		c.437T>A	P	E-4	122Val→Glu	Decreased plasma half-life
16	Komagome-2		c.455A>G	P	E-4	128His→Arg	
17	Asola		c.491A>G	D/P	E-5	140Tyr→Cys	20-25% variant
18	Korea		c.593A>T	D	E-5	174Lys→Ile	
19	Hawkes Bay		c.602G>T	D/P	E-5	177Cys→Phe	5% variant. Decreased α-helical content. Decr eased plasma half-life
20	Ilam		c.643G>A	D/P	E-6	191Ala→Thr	Identified by a new high resolution on-line reverse phase time-of-flight mass spectrometry procedure.
21	FDH-3		c.724C>A	D	E-7	218Arg→Ser	High free T4 andT3. Identified in a Canadian family of Bangladeshi extraction.

22	FDH-1		c.725G>A	D	E-7	218Arg→His	High T4 binding. Low warfarin binding.The most common causal variant in Caucasians.
23	FDH-2		c.725G>C	D	E-7	218Arg→Pro	High T4 binding. Low warfarin binding. Identified in Japanese and Swiss subjects.
24	FDH-4		c.737G>T	D	E-7	222Arg→Ile	High T4 binding. Identified in three unrelated African (Somali) families and one East European (Croatian) family.
25	Tradate-2	Vera Cruz	c.745A>C	D/P	E-7	225Lys→Gln	
26	Herborn		c.790A>G	P	E-7	240Lys→Glu	
27	Skaane		c.875A>G	D/P	E-8	268Gln→Arg	
28	Niigata	Nagasaki-1	c.878A>G	P	E-8	269 Asp→Gly	High prostaglandin binding
29	Caserta		c.900G>C	D/P	E-8	276 Lys→Asn	60-70% variant
30	Tagliacozzo	Cuneo, Cooperstown, Canterbury, New Guinea, Reading, IRE-1, Sweden	c.1011G>T	D/P	E-8	313 Lys→Asn	Low drug binding. Low thermal stability. High progesterone binding
31	Bergamo		c.1013A>G	P	E-8	314 Asp→Gly	
32	Brest		c.1013A>T	P	E-8	314Asp→Val	High fatty acid binding
33	Orebro	Malmo-4	c.1026C>G	D/P	E-8	318 Asn→Lys	
34	Redhill		c.[67C>T (=Malmö-I); 1030G>A]	D/P	E-1; E-8	–2Arg→Cys (=Malmö-I); 320 Ala→Thr;	High fatty acid binding

						N-glycosylated at 318Asn (AsnTyrThr)	
35	Roma		c.1033G>A	P	E-8	321Glu→Lys	Low testosterone binding
36	Sondrio		c.1069G>A	P	E-9	333Glu→Lys	
37	Hiroshima-1		c.1132G>A	P	E-9	354Glu→Lys	
38	Coari I	Porto Alegre I	c.1144G>A	D/P	E-9	358Glu→Lys	
39	Trieste		c.1149G>T/C	P	E-9	359 Lys→Asn	Low thermal stability
40	Parklands		c.1165G>C	P	E-9	365Asp→His	Low drug binding
41	Iowa City-1		c.1166A>T	P	E-9	365Asp→Val	
42	Benkovac		c.1175A>G	D	E-9	368Glu→Gly	
43	Naskapi	Mersin, Komagone-1	c.1186A>G	D/P	E-9	372Lys→Glu	
44	Nagasaki-2	Passo Fundo	c.1195G>A	P	E-10	375 Asp→Asn	
45	Milano slow		c.1195G>C	D/P	E-10	375Asp→His	
46	Tochigi		c.1198G>A	P	E-10	376Glu→Lys	
47	Malmo-3		c.1198G>C	D/P	E-10	376Glu→Gln	
48	Hiroshima-2		c.1216G>A	P	E-10	382Glu→Lys	
49	Liprizzi		c.1300C>T	D/P	E-11	410 Arg→Cys	High S-nitrosylation
50	Dublin		c.1507G>A	P	E-12	479Glu→Lys	
51	Casebrook	Besana Brianza	c.1552G>A	D/P	E-12	494 Asp→Asn; N-glycosylated at 494Asn (AsnGluThr)	High fatty acid binding. Decreased α-helical content
52	Vancouver	Birmingham, Adana, Porto Alegre II, Manaus I, Lambadi, Kashmir, Fortaleza	c.1573G>A	D/P	E-12	501Glu→Lys	
53	Ortonovo		c.1585G>A	P	E-12	505Glu→Lys	
54	Lyon		c.1601A>G	D/P	E-12	510His→Arg	
55	Maddaloni		c.1669G>A	P	E-13	533Val→Met	
56	Castel di Sangro		c.1678A>G	P	E-13	536Lys→Glu	
57	Wuxi		c.1684A>G	D	E-13	538Lys→Glu	
58	South Pacific (see also residue 546)		c.1690A>G	D/P	E-13	540 Thr→Ala	
59	Maku, (Wapishana)	Oriximina I	c.1693A>G	D/P	E-13	541Lys→Glu	High fatty acid binding. High thermal

							stability
60	South Pacific		c.1708G>T	D/P	E-13	546 Ala→Ser	
61	Mexico		c.1721A>G	D/P	E-13	550 Asp→Gly	
62	Dalakarlia-2	Malmö-62	c.1721A>C	D/P	E-13	550Asp→Ala	
63	Church Bay		c.1750A>G	D/P	E-13	560Lys→Glu	
64	Fukuoka-1	Ube-1, Varese-2, Paris-2	c.1759G>A	D/P	E-13	563 Asp→Asn	High fatty acid binding. Decreased α-helical content
65	Osaka-1		c.1765G>A	D/P	E-13	565Glu→Lys	
66	Bazzano		c.1771delT	D/P	E-13	(567)CFAEEGKKLVAASQAALGL (585) → (567)ALPRRVKNLLLQVKLP(582)	High fatty acid binding. Decreased α-helical content
67	B	Oliphant, Ann Arbor, Verona, Osaka-2, Phnom Penh, Nagano, London (Ontario), Lübeck, Tokyo-1, Shinanomachi-1, Iowa City-3, Mayo (MT610, RW246 and SH420), Victoria (East India), Saitama-1, Sweden, Amsterdam	c.1780G>A	D/P	E-13	570Glu→Lys	Low thermal stability
68	Rugby Park		c.1785+1G>C	D/P	I-13	(572)GKKLVAASQAALGL (585) → (572)LLQFSSF(578)	8% variant. High fatty acid binding
69	Banks Peninsula		c.1786-15T>A	D/P	I-13	(572)GKKLVAASQAALGL (585) → (572)SLCSG(576)	
70	Milano fast (Mi/Fg)	Krapina	c.1789A>G	D/P	E-14	573Lys→Glu	This variant was recently

							identified in a 4-year-old Yemeni girl with growth hormone deficiency.
71	Vanves		c.1794A>T/C	D/P	E-14	574 Lys→Asn	
72	Kénitra		c.1794dupA	D/P	E-14	(575)LVAAS QAALGL (585) →(575)TCCC KSSCLRLITS HLKASQPTM RIRERK(603), 2 new SS bonds, T596 is half O-glycosylated	15% variant. Low thermal stability
73	Catania (Ge/Ct)		c.1810delC	D/P	E-14	(580)QAALG L(585) → (580)KLP (582)	
74	Venezia		c.1786_1814+1 delinsAAAAT	D/P	E-14/ I-14	(572)GKKLV AASQAALGL (585) → (572)PTMRIR E (R)(K)(580)	30% variant. Low thermal stability. Increased α-helical content. Decreased plasma half-life

A general feature of the variants modified close to the normal N-terminus (#1-8) is a low affinity for metal ions such as Cu^{++} and Ni^{++}. For additional effects of a propeptide on the structure, stability and function on Alb A, see text.

[a]The variants have usually been named after the place from where the first detected carrier originates.

[b]Codon numbering according to HGVS rules and based on the cDNA sequence NM_000477.7. For converting the present numbering to that of Minghetti et al. [18], see [14].

[c]The structural changes have been determined by DNA (D) or protein (P) sequence analysis.

[d]The positions of proalbumin are from –6 to –1 (the juxtaposition to albumin itself), and those of the mature albumin molecule (Alb A) are from 1 to 585. Addition of 24 to these numbers converts them to a number according to HGVS rules, which is based on the preproalbumin sequence (NCBI Reference Sequence: NP_000468.1).

Table S2. Variants causing analbuminaemia

# [a]	Mutation name [b]	Base change [c]	Intron/ Exon	Protein change [d]	Length of the putative protein product	1st author & year of 1st report	Number of cases
1	Afula	c.1A>C	E-1	undefined defect	?	Caridi-2013	2 closely related families
2	Baghdad	c.79+1G>A	I-1	undefined splicing defect	?	Campagnoli-2002	1
3	Madeira	C.138-2A>G	I-2	undefined splicing defect	?	Caridi-2018	1
4	Codogno	c.166C>T	E-3	p.Gln56*	31	Watkins-1994	1
5	Kayseri	c.228_229delAT	E-3	p.Val78Cysfs*2	54	Galliano-2002	14
6	Amasya	c.229_230delTG	E-3	p.Val78Cysfs*2	54	Caridi-2012	1
7	Treves	c.270+1G>T	I-3	undefined splicing defect	?	Caridi-2016	1
8	Bethesda	c.412C>T	E-4	p.Arg138*	113	Watkins-1994	3 (unrelated)
9	Erzurum	c.527delC	E-5	p.Pro176Argfs*65	239	Caridi-2016	2 (same family)
10	Zonguldak	c.597T>A	E-5	splicing defect?	?	Caridi-2008	1
11	Nijmegen-2	c.615G>A	E-5	undefined splicing defect	?	Unpublished	1
12	Vancouver	c.714-2A>G	I-6	undefined splicing defect	?	Watkins-1994	1
13	Seattle	c.714G>A	E-7	p.Trp238*	213	Rufner-1988	1
14	El Jadida	c.802G>T	E-7	p.Glu268Ter	243	Campagnoli – 2005	2 unrelated
15	Roma	c.872dupA	E-8	p.Asn291Lysfs*8	273	Watkins-1994	2 (same family)
16	Bologna	c.920delT	E-8	p.Leu307Argfs*23	304	Dagnino-2010	1
17	Ghazaouet	c.1098dup	E-9	p.Val367fs*12	353	Caridi – 2019	2 (same family)
18	Roma-2	c.1225C>T	E-10	p.Gln409*	384	Campagna-2005	1
19	Monastir	c.1275C>A	E-10	p.Tyr425*	400	Caridi-2009	1
20	Guimarães	c.1289+1G>A	I- 10	p.Phe398Alafs*33	405	Caridi-2012	5 (2 same family + 3

							unrelated)
21	Hama	c.1309A>T	E-11	p. Lys437Ter	412	Caridi-2020	1
22	Fondi	c.1427A>G	E-11	p.Tyr476Serfs*13	463	Campagna-2005	1
23	Tripoli	c.1428+1 G>T	I-11	undefined splicing defect	?	Bibi-2012	2 (same family)
24	Bartin	c.1428+2T>C	I-11	p.Leu431Tyrfs*5	410	Dolcini-2007	1
25	Tubingen	c.1525C>T	E-12	p.Arg509*	484	Ruhoff-2010	2 (same family)
26	Locust Valley	c.1610delT	E-12	p.Ile537Asnfs*21	532	Davis-2008	1
27	Safranbolu	c.1614_15delCA	E-12	p.Leu540Phefs*2	516	Dagnino-2010	2
28	Ankara	c.1652+1G>A	I-12	p.Leu477Cysfs*4	479	Caridi-2014	1

[a]The mutations are ordered on the basis of their positions in the albumin gene

[b]The mutations have been named after the place from where the first detected carrier originates.

[c]bp and codon numbering is according to HGVS rules and is based on the cDNA sequence NM_000477.7.

[d]The protein changes in analbuminaemia are deduced from mRNA (Guimarães , Fondi, Bartin and Ankara) or DNA (all the others) sequence and were not established at the protein level. Subtract 24 from amino acid numbers to convert to starting at the Asp1 of mature albumin.
